# Effect of digital detox program on electronic screen syndrome among preparatory school students

**DOI:** 10.1002/nop2.1472

**Published:** 2022-11-14

**Authors:** Sayeda Mohamed Mohamed, Lamiaa Saad Abdallah, Fatma Nagy Kotb Ali

**Affiliations:** ^1^ Psychiatric Mental Health Nursing, Faculty of Nursing Cairo University Cairo Egypt; ^2^ Community Health Nursing, Faculty of Nursing Cairo University Cairo Egypt; ^3^ Psychiatric Mental Health Nursing, Faculty of Nursing El Mania University Cairo Egypt

**Keywords:** digital detox programme, electronic screen syndrome, preparatory school students

## Abstract

**Aim:**

The aim of this study was to determine the digital detox programme's impact on the electronic screen syndrome among preparatory school students.

**Design:**

A quasi‐experimental pre‐ and posttest group was used.

**Methods:**

Two preparatory governmental schools. Sample: purposive sample consists of 105 students. Tools: Two tools used for data collection: Student's datasheet and Electronic Screen Addiction Scale. The data collection period took six months, from September 2021 to February 2022.

**Results:**

The high rate of screen addiction among students dropped to 14.3% in the posttest compared with 20.0% in the pre‐test. Moreover, the students' proportion with moderate screen addiction dropped from 65.7% on the pre‐test to 43.8% on the posttest. Furthermore, screen addiction students with lower levels were about 41.9% in the posttest and 14.3% in the pre‐test.

**Conclusions:**

There was a highly statistically significant difference between school students' total electronic screen scores in the pre‐ and posttest. A preventive care programme is recommended for high school children and helps raise screen addiction's awareness and its negative consequences.

**Patient or Public Contribution:**

No patient or public contribution.

## 1 INTRODUCTION

School students are our greatest asset and our hope for the future. Moreover, the stage of childhood represents the foundation for future life. Adolescence is the phase of life, extending between childhood and adulthood; Egypt's population of young people is growing dramatically. About 17 million teenagers (10 to 19 years old) make up roughly 19% of the population (UNICEF Egypt, n.d.).

Electronic screens have increased widely among all people without any age barriers. Devices with screens (TV, Smart Phone, Tablet and PC) that enable us to reach various multimedia have become the essential tools in our daily life, especially for children. In the case of teens, they mainly use mobile phones, and when they get bored with mobiles, they will change to television or laptops. Now, life without screens is not even imagined for teens and adults (Lin et al., 2020).

Children's and adolescents’ lives are increasingly dominated by electronic screen media (ESM). Teenagers use media to learn, communicate, obtain information and seek social support, self‐expression and amusement. Screens have become an uncontrollable fact among people, affecting their daily style, such as their social relationships, physical health, emotional well‐being and productivity (World Health Organization, 2014). Recently, students’ social interactions have been limited due to the COVID‐19 pandemic that has increased; as a result, it leads to increasing the time they spend with multiple screens (Phones, Tablets, PC and TV) and becoming addicted to these devices (Montag & Elhai, 2020).

Electronic screen addiction (ESA) is defined as a repetitive habit pattern towards electronic screens such as smartphones, televisions, laptops and Tablets. That raises the danger of sickness and related emotional and societal problems, commonly referred to as “loss of control.” The overall usage of the electronic screen is high because of a habit known as the “checking habit,” which is the habit of checking their electronic screens more frequently, leading to interference with other aspects of everyday life (Saral & Priya, 2021).

The inability to adjust one's mood, attention or degree of arousal due to engaging with screen gadgets over‐stimulates the youngster and puts the nervous system into fight‐or‐flight mode, resulting in electronic screen syndrome (ESS). The response can be more subtle, as in texting repeatedly, or rapid, as in action games (Hall, 2020).

The time spent in front of an electronic screen, such as televisions, iPads, video games, computers, cell phones, tablets, e‐readers and laptops, is referred to as screen time. Mobile phones, iPads and handheld video game players account for 20% of media consumption. In those 7 h per day, today's youth consume 10 h and 45 min worth of media content, and teenagers devote >11 h daily. Although television and video games are the most common devices, smartphones and tablets gain widespread (Balhara et al., 2018).

One of the essential indicators of electronic screen syndrome is that restricting access to an object creates discomfort when using an electronic screen. Long periods spent staring at an electronic screen led to distress, low self‐esteem, substance abuse, no interest and enjoyment in activities, or feeling hopeless. Electronic screen addiction may have several negative physical, psychological, behavioural, social and health consequences on students and their scholastic achievement (Sarojini et al., 2019).

Electronic media consumption can have a detrimental impact on children's academic achievement, behaviour and health. Teenagers spend a statistically significant amount of time on their smartphones, watching violent television shows or playing violent video games. Electronic screen addiction has also developed into a severe problem with psychological and physiological effects. Furthermore, increased smartphone usage has been linked to insomnia and adverse mental and physical health effects (Chen et al., 2019).

On the contrary today, video games are the most popular entertainment for children and adults globally. Video games are games played using electronic devices such as computers, smartphones or gaming consoles. Video game addiction can occur due to excessive use for an extended period. Teenagers who play violent video games excessively are more prone to fight with their friends, disagree with their teachers and have lower educational levels. Statistics suggest that millions of people enjoy video games, although they have more disadvantages (Almalki & Aldajani, 2021).

The community health nurse has an essential role in reducing electronic screen addiction among school students. As multidisciplinary team members, school health nurses assess, plan, coordinate and implement training programmes for students to help them identify the causes, challenges, effects and solutions for electronic screen addiction. In addition, they clarify the best methods to use electronic screens wisely among adolescents to prevent their negative consequences (Sofia Libriani, 2018).

### 1.1 The significance of the study

Teenagers are the most users of electronic screens. A study conducted in Egypt revealed that 69.6% of preparatory students out of 333 students use electronic screens daily (Nafee et al., 2018). Also, the study revealed that 46.3% of students are highly addicted to their smartphones.

In addition, a study conducted in Egypt to assess the Internet and Facebook addiction among Egyptian students has revealed that students are at an increased risk of problematic Internet use and, to a lesser extent, Internet addiction which negatively affects their academic grades (Saied et al., 2016). Variation in the prevalence of electronic screen addiction among adolescents has been indicated in many studies.

Nevertheless, it was found that many Egyptian researchers have assessed the prevalence of electronic screen addiction among children and its psychological impact. Nevertheless, little Egyptian research was carried out to develop an educational programme to improve the knowledge and practices of younger students about the use of electronic screens wisely. Furthermore, several studies recommend more research about electronic screen syndrome, especially for school students.

Therefore, the current study results will contribute to this aspect and have a statistically significant impact on school nurses applying educational programmes that improve the knowledge and practices of school students about electronic screen syndrome.

This study aimed to assess the effect of a digital detox programme on the electronic screen syndrome among preparatory school students.

### 1.2 Research hypothesis

H: Preparatory students who participated in the digital detox programme would lower electronic screen syndrome levels postprogramme than pre‐programme.

## 2 MATERIALS AND METHODS

### 2.1 Research design

A quasi‐experimental group pre‐ and posttest design was used in this study.

### 2.2 Settings

The study was conducted at two preparatory governmental schools in Cairo governorate which were selected randomly among 32 educational departments. The selected schools were AL Saniya preparatory and AL‐Nasr preparatory governmental language schools. These schools represented Misr EL Kadima and EL Sayeda Zeinab educational departments. Each school contains classes from first to third preparatory grades. The first school consists of 4 buildings, 15 classes approximately, and is equipped with computer laboratories with data show and whiteboard, while the other consists of 3 floors and five classes on each floor.

### 2.3 Sample

The study included a purposive sample consisting of 105 preparatory students. A sampling technique was used: One school was selected randomly from the educational department in the South of Cairo. After selecting two schools, two classes from each school were randomly selected from a 1st to 3rd‐grade list. The statistical analyser determined the number of 105 preparatory students. Sample size was calculated by using the Cochran formula for sample size with margin of error taken to be 8% and the population size was 355 (Som, 1996).

$$n=\frac{z^{2}*p*\left(1-p\right)/e^{2}}{1+\frac{z^{2}*p*\left(1-p\right)}{e^{2}*N}}$$

The students were selected, particularly as adolescence was an appropriate age for implementing the educational programme.

### 2.4 Inclusion criteria

Students aged 13–15 years of both sexes, who might have smartphones or tablets and spent most of their time on electronic screens (more than 2 h) according to the guidelines prescribed by the American Academy for Pediatrics (2016) on the use of smart devices, were included in the study.

### 2.5 Tools

Data were collected through three tools:

#### 2.5.1 Student's datasheet

It was developed by the researcher and included two parts:

*1st part*: It included demographic data and their parents. It consisted of seven questions: age, sex, grade, parent's educational level and occupation. This part was used only before the application of the educational programme.

*2nd part (student's general information related to electronic screen)*: It was used to assess students’ daily living activity on electronic screens. This part consisted of six questions related to the most used electronic devices, the number of hours spent on social media, watching TV or videos and playing video games. It was used as a pre‐test only. The questions were formulated as multiple‐choice questions with no correct or incorrect options. The student's responses reflected his/her point of view.

#### 2.5.2 Electronic Screen Addiction Scale

It was developed by the researchers after reviewing the relevant national and international literature. It was designed based on the study conducted by Saritepeci (2021). The developing tool was used to assess electronic screen addiction among preparatory school students. It consisted of 27 items. The scale was divided into three domains: excessive screen time (5 items), compulsive behaviour (15 items) and loss of control (12 items). The Arabic version of the electronic screen addiction scale was tested for content validity and revised by expert psychiatric nurses.

The tool was on a three‐point Likert scale, ranging from strongly agree = 3, neutral = 2, to disagree = 1. Total scale scores ranged from 27 to 81. High scores showed a high level of electronic screen addiction among preparatory students. The following scores were used to determine the levels of addiction for electronic screens: 60.75–81 (≥75%), moderate addiction scored from 40.5–60 (50% to <75%), less than 40.5 (<50%) was considered non‐addicted. Cronbach's Alpha was used to determine the internal consistency of the developed tool, which was (0.774).

### 2.6 Ethical considerations

Official approval was obtained from the educational directorate, the educational zones and the headmaster in AL Saniya preparatory school for girls and AL‐Nasr preparatory governmental language school. Participation in the study was voluntary and based on parents and child acceptance. Informed consent was obtained from all parents who agreed to participate in the study. Data confidentiality and students' privacy were secured. Oral consent was obtained from the children to participate in the study. The researchers created code numbers and kept them to keep the students anonymous. The study's purpose was explained to students to gain their cooperation and facilitate the data collection process. Data collection lasted for 2 months. The study was carried out in four phases: assessment phase, planning, implementation and evaluation. The duration of each session was about 30 min. The researcher introduced the programme to each class in the same manner. All students in the selected classes were enrolled in the programme sessions when fulfilling the selection criteria.

#### 2.6.1 Programme description

Structured nursing interventions were designed for digital detox strategies (Internet resources, journals and nursing textbooks). This programme aimed to assess the effect of the digital detox programme on electronic screen syndrome among preparatory school students. The programme consisted of four phases. The assessment phase: The evaluation was done using a screen addiction scale to evaluate electronic screen addiction among students. The time spent filling out the questionnaire ranged from 15–25 min. Planning phase: The researcher designed the digital detox programme. This digital detox programme aimed to reduce electronic screen syndrome among preparatory school students. The number of students in each theoretical and practical session was delineated. The school administration also decided the place and time of sessions. Teaching methods and media were planned during this phase.

##### Implementation phase (4 sessions)

The programme was divided into four sessions, with each session consisting of a teaching class using pre‐designed training materials.

At the beginning of each session, an orientation to the session objectives occurred. Feedback was given at the end of each session. Teaching methods used during the programme implementation were lectures, discussions and demonstrations. Also, educational media used during the programme was a whiteboard. Supporting materials for each session, such as pictures, were used.

The first session was introductory, where the researcher built a rapport and a relaxing atmosphere with the school children and told the student about the programme and the aim and questionnaires. The first session included a simple explanation of electronic screen addiction and students' background about the programme.

The second session included information about the electronic syndrome, explaining screen addiction and the types of electronic screens, such as television, smartphones, computer and electronic games.

The third session included the negative consequences of electronic screen exposure and prevention methods to reduce screen time and limit screen exposure.

The fourth session included signs and symptoms of electronic screen addiction and the relationship with scholastic achievement. Feedback was taken and given during each session. A booklet enriched with illustrative pictures was distributed at the end of the educational programme to reference the children.

##### Evaluation phase

In this phase, the digital detox programme evaluation was done using the electronic screen syndrome scale postdigital detox programme to assess the achievement of intervention objectives. One month later, it was followed up to ensure the stability of knowledge and practice.

**Table:** 

Stage	Content	Duration
Assessment phase	Assessment of electronic screen addiction among students using screen addiction scale	1 month
Planning phase	The researcher designed the digital detox programme. This digital detox programme aimed to reduce electronic screen syndrome among preparatory school students The number of students in each theoretical and practical session was delineated The school administration also decided the place and time of sessions. Teaching methods and media were planned during this phase	1 month
Implementation	In this phase, the educational programme was conducted and presented in simple Arabic language The designed programme was carried out in four sessions: each session was given in the form of teaching class through pre‐designed training materials Teaching methods used during the programme implementation were lectures, discussions and demonstrations. Also, educational media used during the programme was a whiteboard. Supporting materials for each session, such as pictures, were used	1 month
Programme session	The first session was introductory, where the researcher built a rapport and a relaxing atmosphere with the school children and told the student about the programme and the aim and questionnaires. The first session included a simple explanation of electronic screen addiction and students' background about the programme The second session included information about the electronic syndrome, explaining screen addiction and the types of electronic screens, such as television, smartphones, computer and electronic games The third session included the negative consequences of electronic screen exposure and prevention methods to reduce screen time and limit screen exposure The fourth session included signs and symptoms of electronic screen addiction and the relationship with scholastic achievement. Feedback was taken and given during each session. A booklet enriched with illustrative pictures was distributed at the end of the educational programme to reference the children	
Evaluation	In this phase, the digital detox programme evaluation was done using the electronic screen syndrome scale postdigital detox programme to assess the achievement of intervention objectives	2 month

### 2.7 Pilot study

The pilot study was done on 10% of school children to examine the study feasibility, clarity of questions and time needed to complete the study. No modifications were made based on the results, and school children who participated in the pilot study were included in the actual study.

### 2.8 Statistical design

The information gathered from the children was coded and analysed using the Statistical Package for the Social Sciences (SPSS) version 20. Descriptive statistics such as percentage, mean and standard deviation were used to analyse the data. ANOVA test was done to answer the main research question and also, to find the effect of some independent variables on the respondent's practice in pre‐test, posttest and follow‐up test. The statistically significant level of all statistical analyses was .05 (*p*‐value). The *p*‐value >.05 indicates insignificant result. The *p*‐value <.05 indicates statistically significant result. *t*‐test was used to determine the statistically significant differences between all electronic screen dimension scores among pre‐ and posttest.

## 3 RESULTS

Table 1 reveals that 46.7% of school students aged from 13 to less than 14 years old, and only 5.7% were ≥15 years old. The mean age was 13.4952 and SD ±0.68112. Furthermore, 71.4% of students were female, and 28.6% were male. 74.3% of them were in the second preparatory educational grade.

**TABLE 1:** Percentage distribution of the school students about age, sex and educational grade (*n* = 105)

Variables	*N*	%
*Age*		
12 to <13	5	4.8
13 to <14	49	46.7
14 to <15	45	42.9
≥15	6	5.7
*Sex*		
Male	30	28.6
Female	75	71.4
*Educational grade*		
1st preparatory	27	25.7
2nd Preparatory	78	74.3

**FIGURE 1:**
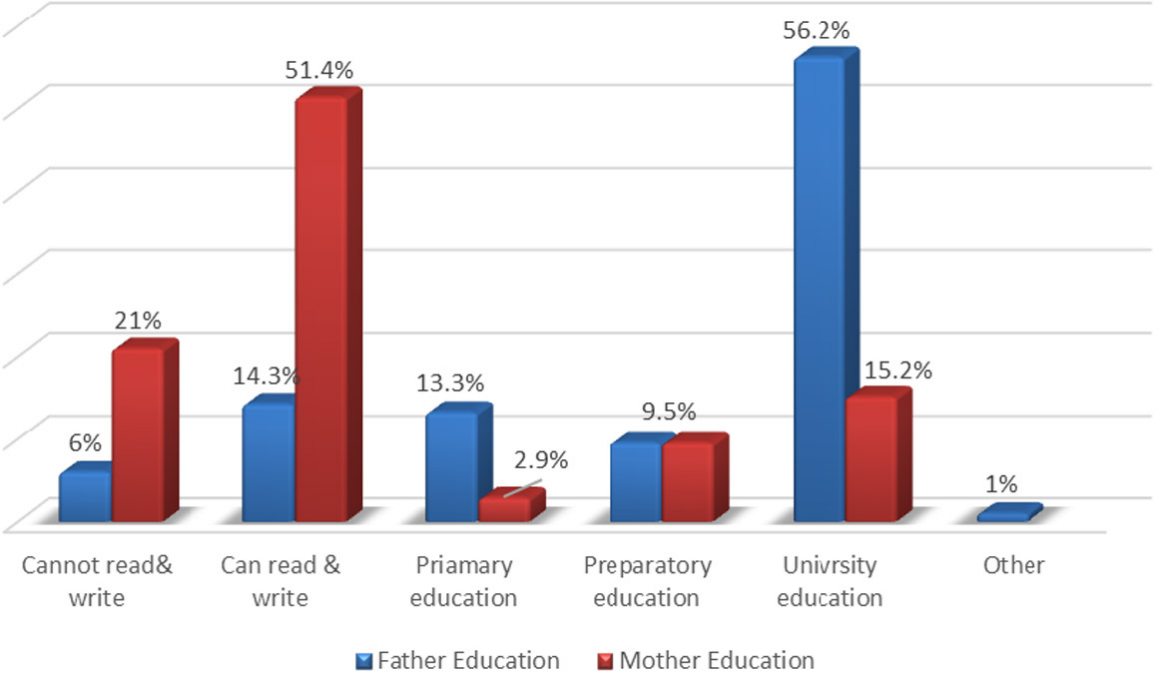
Percentage distribution of the students regarding parent's educational level (*n* = 105)

Concerning parents' educational levels, Figure 1 illustrates that 56.2% of the fathers had a university education, while 51.4% of students' mothers could read and write.

**FIGURE 2:**
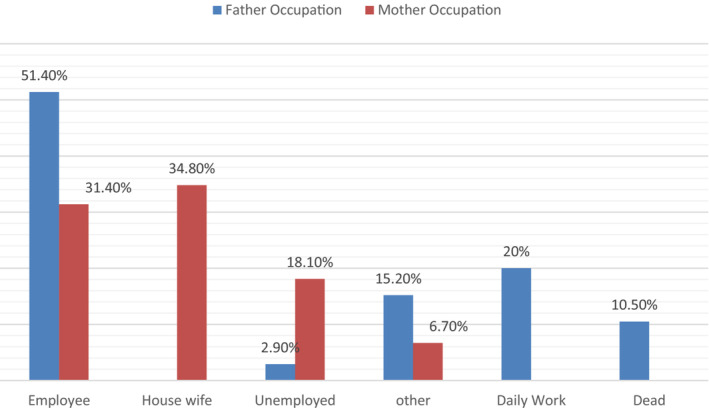
Percentage distribution of the students regarding parents' occupation (*n* = 105)

Figure 2 shows that 43.8% of fathers and mothers of school students were employees and housewives, respectively, concerning the parent's occupation.

**FIGURE 3:**
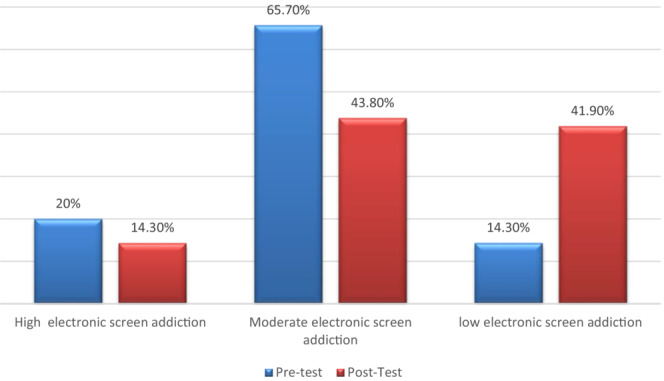
Percentage distribution of the students regarding electronic screen addiction level before and after the programme (*n* = 105)

Figure 3 indicates an improvement in the posttest results compared with the pre‐test. The percentage of students with high electronic screen addiction decreased to 14.3% in the posttest compared with 20.0% in the pre‐test. In addition, the percentage of students who had moderate electronic screen addiction decreased from 65.7% in the pre‐test to 43.8% in the posttest. Moreover, the students with low electronic screen addiction recorded about 41.9% in the posttest compared with 14.3% in the pre‐test**. This figure supports the research hypothesis.**

Table 2 shows high statistically significant differences between all electronic screen dimension scores among pre‐ and posttest. Also, there was a highly statistically significant difference between the total electronic screen scores of the school students in pre‐test and posttests (*p*‐value = .000). This table supports the research hypothesis.

**TABLE 2:** Difference between the mean scores of electronic screen addiction dimensions in pre‐ and posttests

Dimensions	Pre‐test		Posttest		*t*	*p*
	Mean	SD	Mean	SD		
Total excessive screen time	10.63	.466	9.22	2.97	349	.000*
Total compulsive Behaviour	28.87	.149	25.27	7.34	.313	.001*
Total loss of control	11.70	2.48	10.55	3.14	.388	.000*

* The mean difference is statistically significant at the level of 0.05.

## 4 DISCUSSION

Despite the many positive impacts of Internet use, it has become more troublesome and linked to decreased physical activity and various detrimental neurophysiological and psychosocial repercussions in adolescents. Heavy usage in vulnerable teens can develop into an addiction, and it is a growing public health concern with varying incidence rates (from 0.8% in Italy to 26.7% in Hong Kong) (Dreier et al., 2015).

This finding contradicts the findings of (Su et al., 2019), who found that gender might have varied effect sizes, both in magnitude and directionality, for certain behaviours, including gaming (where males might exhibit more **IA** symptoms) and social networking (where a female could show higher symptoms). Regarding the educational grade, most of them were in the second preparatory educational grade.

Regarding the electronic screen addiction level before and after the programme, the present study illustrated that the percentage of students with high and moderate electronic screen addiction decreased in the posttest more than in the pre‐test. In addition, students with low electronic screen addiction increased in the posttest than in the pre‐test (Figure 1). These results might be due to the impact of the digital detox programme, which increased students' awareness of the advantages and disadvantages of technology and the physical and psychological impact of exposure to electronic screens. Also, the development of communication skills improved their abilities towards engagement in life attitudes and value orientations, which were necessary to prevent dependence on an electronic screen. All of these techniques helped decrease their use of electronic screens.

These findings were consistent with those of (Bubnova et al., 2018), who claimed that Internet addiction among students decreased due to their work. The following are quantifiable markers of these changes: The number of young individuals who had a high level of education was not disclosed. In the experimental group, the number of students with an average level decreased by 14%, while it dropped by 4% in the control group. In the experimental group, the number of students with a low level of Internet addiction grew by 24%, while in the control group, it increased by 4%.

Regarding the difference between the mean scores of electronic screen addiction dimensions in pre‐ and posttests, Table 2 demonstrates high statistically significant differences between all electronic screen dimensions (total excessive screen time, total compulsive behaviour and total loss of control) scores among school students in pre‐ and posttests. Also, there was a highly statistically significant difference between total electronic screen scores of the school students in pre‐ and posttests. These results might be related to the programme's positive impact as those students avoided real‐life communication, adopted destructive ideas in some content and spent excessive time participating in virtual games. After the programme application, it provided students with alternative activities, strengthening teens’ resources that could inhibit the development of self‐destructive conduct, parental control over the student's daily schedule and advised work with parents.

This finding was in line with those (Yang & Kim, 2018), who discovered that all interventions had substantial impacts in the desired direction. After completing the programme, statistically significant changes were found to considerably reduce IA symptoms in the intervention group compared with the control group. Unlikely, it contradicted (Yang et al., 2020), who claimed that after the health behaviours intervention, a statistically significant decrease in the proportion of students with high screen time and IA symptoms was detected.

## 5 CONCLUSIONS

Based on the current findings, preparatory students had a high electronic screen addiction level before the programme, which decreased after the programme. There was a highly statistically significant difference between total electronic screen scores of the school students in pre‐ and posttests. There was a highly statistically significant correlation between school students' total electronic screen scores and parents' education and occupation.

### 5.1 Recommendations

- Preparatory students should limit their usage of electronic displays to avoid addiction and maintain their daily priorities.
- Preventative nursing programmes must be developed for high school children, and awareness should be raised about electronic screen addictions and their negative consequences.
- Effective counselling programmes should be developed to prevent electronic screen addiction.
- Awareness among preparatory students must be raised about the physical, psychological and social hazards of electronic screen addiction.

## AUTHOR CONTRIBUTIONS

All authors contributed to the conception and study design, data collection, analysis, interpretation, manuscript writing, reviewing and revising. All authors read and approved the final manuscript.

## CONFLICT OF INTEREST

Sayeda, M. Mohamed Lamiaa S. Abdallah and Fatma N. Ali declare no potential conflicts of interest with respect to the research, authorship or publication of this article.
